# Alteration of Gene Expression Profile in Niemann-Pick Type C Mice Correlates with Tissue Damage and Oxidative Stress

**DOI:** 10.1371/journal.pone.0028777

**Published:** 2011-12-22

**Authors:** Mary C. Vázquez, Talía del Pozo, Fermín A. Robledo, Gonzalo Carrasco, Leonardo Pavez, Felipe Olivares, Mauricio González, Silvana Zanlungo

**Affiliations:** 1 Departamento de Gastroenterología, Facultad de Medicina, Pontificia Universidad Católica de Chile, Santiago, Chile; 2 Laboratorio de Bioinformática y Expresión Génica, INTA, Universidad de Chile, Santiago, Chile; 3 Fundación Hospital Parroquial de San Bernardo, Santiago, Chile; 4 Laboratorio de Bioinformática y Matemáticas del Genoma, Centro de Modelamiento Matemático (CMM), Facultad de Ciencias Físicas y Matemáticas, Universidad de Chile, Santiago, Chile; 5 FONDAP-Center of Genome Regulation (CGR), Santiago, Chile; International Centre for Genetic Engineering and Biotechnology, Italy

## Abstract

**Background:**

Niemann-Pick type C disease (NPC) is a neurovisceral lipid storage disorder mainly characterized by unesterified cholesterol accumulation in lysosomal/late endosomal compartments, although there is also an important storage for several other kind of lipids. The main tissues affected by the disease are the liver and the cerebellum. Oxidative stress has been described in various NPC cells and tissues, such as liver and cerebellum. Although considerable alterations occur in the liver, the pathological mechanisms involved in hepatocyte damage and death have not been clearly defined. Here, we assessed hepatic tissue integrity, biochemical and oxidative stress parameters of wild-type control (*Npc1* ^+/+^; WT) and homozygous-mutant (*Npc1* ^−/−^; NPC) mice. In addition, the mRNA abundance of genes encoding proteins associated with oxidative stress, copper metabolism, fibrosis, inflammation and cholesterol metabolism were analyzed in livers and cerebella of WT and NPC mice.

**Methodology/Principal Findings:**

We analyzed various oxidative stress parameters in the liver and hepatic and cerebellum gene expression in 7-week-old NPC1-deficient mice compared with control animals. We found signs of inflammation and fibrosis in NPC livers upon histological examination. These signs were correlated with increased levels of carbonylated proteins, diminished total glutathione content and significantly increased total copper levels in liver tissue. Finally, we analyzed liver and cerebellum gene expression patterns by qPCR and microarray assays. We found a correlation between fibrotic tissue and differential expression of hepatic as well as cerebellar genes associated with oxidative stress, fibrosis and inflammation in NPC mice.

**Conclusions/Significance:**

In NPC mice, liver disease is characterized by an increase in fibrosis and in markers associated with oxidative stress. NPC is also correlated with altered gene expression, mainly of genes involved in oxidative stress and fibrosis. These findings correlate with similar parameters in cerebellum, as has been previously reported in the NPC mice model.

## Introduction

Niemann-Pick type C disease (NPC) is a neurovisceral atypical lipid storage disorder involving endocytosed cholesterol [1], as well as other kind of lipids. NPC is a fatal autosomal recessive disease caused by mutations in the *NPC1* or *NPC2* gene. The *NPC1* gene encodes a lysosomal transmembrane protein, and the *NPC2* gene encodes a soluble lysosomal protein that binds cholesterol. Both proteins are involved in cholesterol trafficking from lysosomes [2]. Mutations in the *NPC1* gene account for approximately 95% of NPC cases [3]. In the pathology, the deficiencies of these proteins lead to the accumulation of free cholesterol and secondarily glycosphingolipids, such as lactosilceramide, glucosylceramide and GM2 and GM3 gangliosides in the lysosome [4], [5], [6], [7]. NPC is characterized by an inability to process cellular cholesterol from the endocytic pathway, leading to late endosomal-lysosomal accumulation of cholesterol and glycosphingolipids and abnormal tubulovesicular trafficking, progressive neuropathology and neurodegeneration. The disease is often diagnosed in early childhood, with patients typically displaying cerebellar ataxia, difficulty speaking and swallowing, and progressive dementia [1], [3]. These symptoms are associated with damage to the central nervous system (CNS), especially in the cerebellum, where extensive and progressive neuronal death is observed [8].

The CNS is particularly dependent on cholesterol metabolism and is especially sensitive to oxidative stress damage [9]. This sensitivity is mainly due to several features of the CNS: the high concentration of polyunsaturated fatty acids that are susceptible to lipid peroxidation, the relatively large amounts of oxygen consumed for energy production, and the fewer antioxidant defenses available to the CNS compared to other organs. Neurons are particularly vulnerable to oxidative stress because they have low levels of reduced glutathione [10]. Oxidative stress has been shown in NPC mouse brain [11] and in different NPC cellular models [12]; however, its functional relevance to the disease process has not yet been established. Previous data from our laboratory suggest an increase in oxidative stress markers *in vitro* in NPC models and *in vivo* in the cerebellum of *Npc1* ^−/−^ mice [13]. We have also found that in a neuronal model of the disease, treatment with an antioxidant compound prevents cellular death and apoptosis [13]. In summary, our data are in agreement with previous findings suggesting that oxidative stress is central to neuronal NPC pathology.

A risk factor for oxidative stress relevant to nervous tissue is the level of metal ions, such as copper. Copper is an important oligoelement that plays an essential role in human physiology [14]. Appropriate levels of copper are essential to avoid cellular damage by oxidative stress due to the rapid oxidation of copper, which causes damage to the biomolecules listed above and generates ROS, leading to cell death. Copper from the diet is mainly stored in the liver, the organ that regulates the distribution of copper through release into the plasma or excretion via bile. Moreover, the expression of genes involved in metal homeostasis and transport, including iron, copper and zinc were reported to be altered in NPC fibroblast microarray studies [15], [16] suggesting alterations in metal levels in NPC disease.

Although neuronal damage is a major feature of NPC, most patients present considerable damage in the liver [4]. Indeed, currently, Niemann-Pick C disease is recognized as a relatively common cause of liver disease in early life [17]. Liver damage is particularly relevant in NPC disease because it plays a central role in maintaining whole-body cholesterol metabolism. Approximately half of NPC patients suffer from liver disease, and NPC may be the most common metabolic disorder responsible for neonatal cholestasis [3]. As early as the neonatal period, NPC patients may present with neonatal jaundice, hepatosplenomegaly, failure to thrive and death between 3 and 9 months (in the most severe form of the disease) or between 1 and 3 years (most commonly) [1], [3]. The histopathology of the liver resembles neonatal hepatitis, and as the disease progresses, the liver accumulates more storage material, such as lipids and unesterified cholesterol. The pathophysiology associated with NPC liver disease and aspects of lipoprotein metabolism have been investigated using the NPC mouse model. An increase in hepatic cholesterol by dietary administration results in hepatic damage and cellular death [18]. An increase in cellular cholesterol oxidation products has been described in NPC mouse tissues, plasma and macrophages from NPC mice [19], [20] whereas a decrease in the antioxidant capacity has been shown in hepatocytes from human NPC patients [21]. It has also been suggested recently that the cholesterol oxidation products could serve as blood-based biomarkers for the NPC disease [22].

One approach to determine the altered physiological pathways in disease is to analyze gene expression profiles. Several reports have shown alterations in gene expression in fibroblasts of human NPC patients and in the cerebellum of NPC mice [15], [16], [23]. Herein, we performed a histopathological analysis of the liver of *Npc1* ^−/−^ (NPC) mice to evaluate liver damage. We also performed a biochemical analysis of oxidative stress markers and measured total copper content in liver tissue. Finally, we evaluated liver and cerebellum gene expression using qPCR and microarray techniques, focusing on genes related to biological processes that are altered in the disease.

We found that in livers of NPC mice, there was an increase in inflammation reaction and oxidative stress–induced damage in comparison with control animals, as shown by histological analysis, increased biochemical oxidative stress markers, such as protein carbonyls, and decreased antioxidant species, such as reduced glutathione. These observations correlated with differential expression of hepatic genes related to oxidative stress in NPC mice as verified by qPCR analysis. We also analyzed cerebellum gene expression patterns by qPCR and microarray assay and found differential expression of cerebellar genes associated with cholesterol metabolism, inflammation and fibrosis processes in NPC mice. Finally, we performed a preliminary acute intervention with N-acetyl cysteine (NAC), a widely used antioxidant. After this, we observed a decreased level of inflammation in livers of NPC mice.

## Materials and Methods

### Animals and diet

BALB/c mice carrying a heterozygous mutation in the NPC1 gene [24] were used to generate wild-type control (*Npc1* ^+/+^; WT) and homozygous-mutant (*Npc1* ^−/−^; NPC) animals. Genotypes were identified as described previously [25]. All mice had free access to water and chow diet (<0.02% cholesterol; Prolab RMH 3000, PMI Feeds Inc., St. Louis, MO) until they were used for studies. For experiments, 7- and 8-week-old NPC and WT male mice were fasted for 2 hours before liver sampling. Protocols were performed according to the Public Health Service Policy on Humane Care and Use of Laboratory Animals in the Institute for Laboratory Animal Research Guide for Care and Use of Laboratory Animals and approved by the review board for animal studies at our institution (Comité Etica Bienestar Animal, CEBA-MedUC; Approval ID#3-2009).

### Liver and cerebellum sampling

Mice were anesthetized by intraperitoneal injection of ketamine (80–100 mg/kg) and xylazine (5–10 mg/kg). The liver was sectioned into three parts; one part was used for histological analysis (1–1.5 cm long) and stored in 10% formalin solution, a second part (about 500 µg) was used for copper measurement and stored at −80°C, and the remaining tissue was used for RNA extraction and also stored at −80°C. Cerebella were dissected and stored at −80°C. For RNA extraction purposes, tissues were stored in RNAlater solution (Ambion, USA).

### Biochemical assays for oxidative stress markers

#### Protein carbonyls

One hundred milligrams of fresh tissue was disrupted in 3 ml of homogenization buffer (0.1% digitonin and 1 mM EDTA in 50 mM sodium phosphate buffer, pH 7.4) plus proteases inhibitors (5 mg/ml leupeptin, 5 mg/ml pepstatin and 50 mg/ml PMSF). After 15 min at room temperature, the homogenates were centrifuged at 5,000× *g* for 20 min. Aliquots of 1 ml from each sample were incubated either with 4 ml of either dinitrophenylhydrazine (DNPH, 10 mM in 2.5 M HCl) or 2.5 M HCl for blank determination. Tubes were then incubated for 1 hour at room temperature in the dark with vortexing every 15 min. Proteins were then precipitated with trichloroacetic acid (TCA, 10% final concentration), and the pellets were washed with an ethanol∶ethyl acetate (1∶1) solution. The final pellet was resuspended in 2 ml of 6 M urea and incubated for 10 min at room temperature. The amount of protein carbonyls was measured by absorbance in the 350–390 nm range.

#### Total glutathione

Total glutathione content in liver samples was performed as previously described [26]. Briefly, 200 mg of frozen tissue was mechanically homogenized in 2 ml of 5% (w/v) sulfosalicylic acid. The homogenate was centrifuged, and the resulting supernatant was diluted 1∶25 in 5% (w/v) sulfosalisylic acid. A 25-µl aliquot was then incubated with NADPH and DTNB solutions at 37°C for 10 min prior to the addition of glutathione reductase (1.8 units per cuvette). The released product was measured at an absorbance of 412 nm. A calibration curve of reduced glutathione ranging from 20 to 80 µM was performed.

### RNA extraction

Total RNA was extracted from homogenized liver or cerebellum with TRI Reagent (Ambion) according to the manufacturer's instructions. RNA quality and quantity were assessed prior to and after DNase digestion by denaturing gel electrophoresis and photometric analysis (A_260_/_280_ ratio), respectively.

### mRNA synthesis and Fluorescent Labeling

One microgram of total RNA was used to synthesize mRNA using the MessageAmp II mRNA amplification kit (Ambion). Five micrograms of mRNA from WT and NPC mice was coupled with Cy3 and Cy5 dyes, respectively, according to the manufacturer's instructions. Probe quantity and dye incorporation were assessed with a scanning spectrophotometer.

### Microarray Hybridization

Two and five micrograms of each labeled probe was used for hybridization for liver and cerebellum samples, respectively. The two dye probes were mixed and concentrated to a volume of 30 and 50 µl for liver and cerebellum samples, respectively in a solution containing 20% formamide, 5× SSC and 0.1% SDS, and hybridization was performed essentially according to the microarray manufacturer's instructions. Slides were incubated for 16 hours at 42°C as previously described [27], and hybridization was automatically performed using the HybArray 12™ DNA hybridization system (PerkinElmer). For detection of the fluorescent derivatives, we used a ScanArray GX laser reader.

### Data Analysis

The overall expression of genes was performed using a Mouse Ready Array from Microarrays, Inc. (Nashville, TN). Each array contained 35,302 70-mer oligonucleotides, representing ∼25,000 genes. Spot identification and quantification were performed with GenePix 5.1 software (Molecular Devices). Array data were analyzed using the R statistical language and environment (http://www.r-project.org), specifically with the microarray analysis tools available from the Bioconductor Project (http://www.bioconductor.org). Spots that showed qcom<0.5 [28] were considered low-quality spots and were removed. Data were background-subtracted and normalized using the LIMMA Bioconductor package [29]. Data obtained from biological replicates were averaged, and then linear models were applied. Differentially expressed genes were determined using empirical Bayesian methods, and p<0.01 was considered statistically significant [29]. Analysis of enriched gene ontology (GO) categories of differentially expressed genes was performed using the Gene Ontology Tree Machine (GOTM) [30]. The parameters used were the hypergeometric statistical method and a Bonferroni multiple test adjustment, and p<0.05 was considered statistically significant. All data is MIAME compliant and the genes described herein have been deposited in NCBI's Gene Expression Omnibus (GEO, http://www.ncbi.nlm.nih.gov/geo/) and are accessible through GEO number GSE24013.

### Real-Time PCR (qPCR)

Total RNA (2 µg) was used as a template for reverse transcription reactions to synthesize single-stranded cDNA using MMLV-RT reverse transcriptase (Promega) and an oligo (dT) primer according to standard procedures. Gene-specific primer sets (detailed in Table S1) were designed by Primer3Plus to amplify DNA products of 70 and 150 bp. Real-time RT-PCR (qPCR) reactions were performed in a LightCycler (Roche) using SYBR Green to monitor cDNA amplification. A 1∶25 dilution of cDNA was used in each reaction along with 1 µl of FastStart DNA Master SYBR Green I (Roche), 0.8 µl MgCl_2_ (25 mM) and 2.5 pmol of forward and reverse primers in a total volume of 10 µl. The following standard thermal profile was used: 10 min at 95°C, 40 repeats of 10 s at 95°C and 15 s at 60°C, with a final 10-s stage at 72°C. Data were analyzed using LightCycler Software (v.3, Roche). Efficiency was determined for each sample and gene by LinRegPCR v.7.5 using data obtained from the exponential phase of each amplification plot [31]. Two technical replicates were done for each combination of cDNA and primer pair, and the quality of the PCR reactions was determined by analysis of the dissociation and amplification curves. The products were resolved by 3% agarose gel electrophoresis to confirm the DNA fragments of expected size. Transcript levels of genes were normalized to the expression values of the PPIA gene [32], which was validated in our experimental conditions by NormFinder [33]. qPCR was performed in samples from at least three mice, and differences in gene expression levels between WT and NPC mouse liver samples were analyzed by Mann–Whitney U-test. p<0.05 was considered statistically significant.

### Quantification of Cu

Metal content was quantified using 20 mg of liver tissue that was disrupted in 65% concentrated suprapure nitric acid (Merck, Chemical Co., Darmstadt, Germany) for 24 h at 60°C and then diluted to a final concentration of 5%. Cu determination was made using a graphite furnace atomic absorption spectrophotometer (Perkin Elmer, SIMMA 6100). Calibration was against a Cu standard curve (J.T. Baker), and the sample values were normalized to the values of fresh weight (FW). The statistical analysis for cellular Cu content was done by Mann–Whitney U-test, and p<0.05 was considered statistically significant.

### Hepatic histology

Liver tissue was fixed in 4% paraformaldehyde for 48 h and then embedded in paraffin, sectioned, and placed on glass slides. Hematoxylin and Eosin and Van Gieson staining were performed according to standard procedures and were blindly analyzed by a pathologist.

## Results

### Hepatic tissue damage in NPC mice

**Figure 1:**
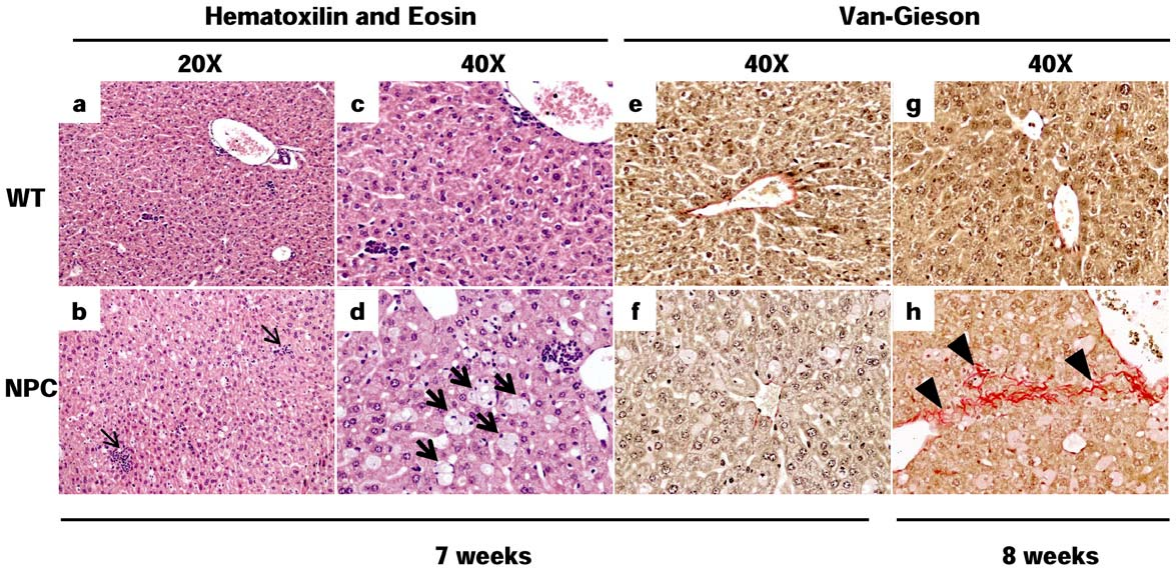
Hepatic tissue damage is increased in NPC mice. Hematoxylin and eosin staining (a–d) to assess tissue integrity and Van Gieson staining for collagen (e–h) in WT (upper panel) and NPC (lower panel) mice are shown. Inflammatory foci (thin arrows), lipid-loaded macrophages (bold arrows) and lobular fibrosis (arrowheads) are indicated.

The liver histology of 7-week-old NPC and WT mice was evaluated by light microscopy (Figure 1). We observed several inflammatory foci in the livers of NPC mice (panel b, thin arrows) that were absent in livers of WT mice (panel a). Furthermore, several cells with a foamy cytoplasm, were observed in NPC livers, indicating lipid accumulation (panel d, bold arrows), but not in livers of WT mice (panel c). Van Gieson staining for collagen was negative in 7-week-old WT and NPC mice (panels e and f); however, 8-week-old NPC mice showed a strong positive mark across the lobule (panel h, arrowheads), indicating that the fibrotic process was actually enhanced. In contrast, 8-week-old WT mice had no evidence of liver fibrosis (panel g).

### Oxidative stress markers and copper levels in livers of NPC and WT mice

**Figure 2:**
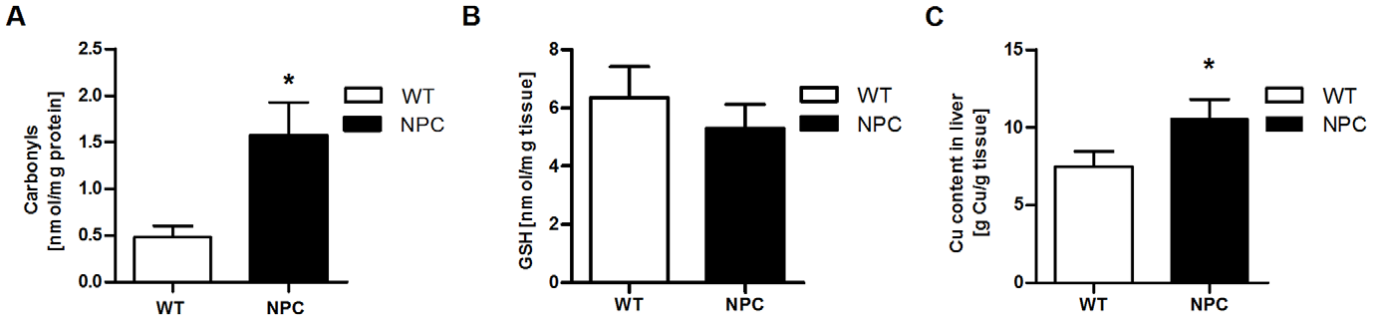
Oxidative stress markers are altered in NPC versus WT mice liver. (A) Carbonyl adducts expressed as nmol/mg of protein in WT and NPC mice liver. Statistical analysis, unpaired Student's t-test, *p<0.05; n = 8 for each group. (B) Levels of reduced glutathione expressed as nmol/mg of tissue in WT and NPC mice. (C) Copper content expressed as µg copper/g fresh weight (FW) in liver tissue of WT and NPC mice. Statistical analysis, Mann–Whitney U-test, *p<0.05; n = 3 for each group.

As an initial search for markers of oxidative stress in NPC mice, we measured hepatic protein carbonyls and reduced glutathione concentration in 7-week-old animals. As shown in Figure 2, protein carbonyls were significantly increased (Figure 2A), while glutathione showed a tendency to decrease (Figure 2B) in livers of NPC mice compared to WT mice. These results suggest that the histological changes observed in NPC mice are correlated with oxidative stress. We next analyzed tissue copper content as a possible indicator of an imbalance in oxidative metabolism in NPC liver. Interestingly, our data indicate a significant increase in copper content in the livers of NPC mice (Figure 2C).

### Gene expression analysis in livers and cerebella of NPC and WT mice

Prior to qPCR analysis of gene expression in livers and cerebella of NPC mice, we analyzed three potential housekeeping genes to use for normalization of the data. These genes were those encoding ribosomal protein L4 (*Rpl4*), TATA-binding protein (*Tbp*) and peptidylprolyl isomerase A (*Ppia*). Based on stability value criteria [34], we chose *Ppia* (Table S2 and Figure S1).

**Figure 3:**
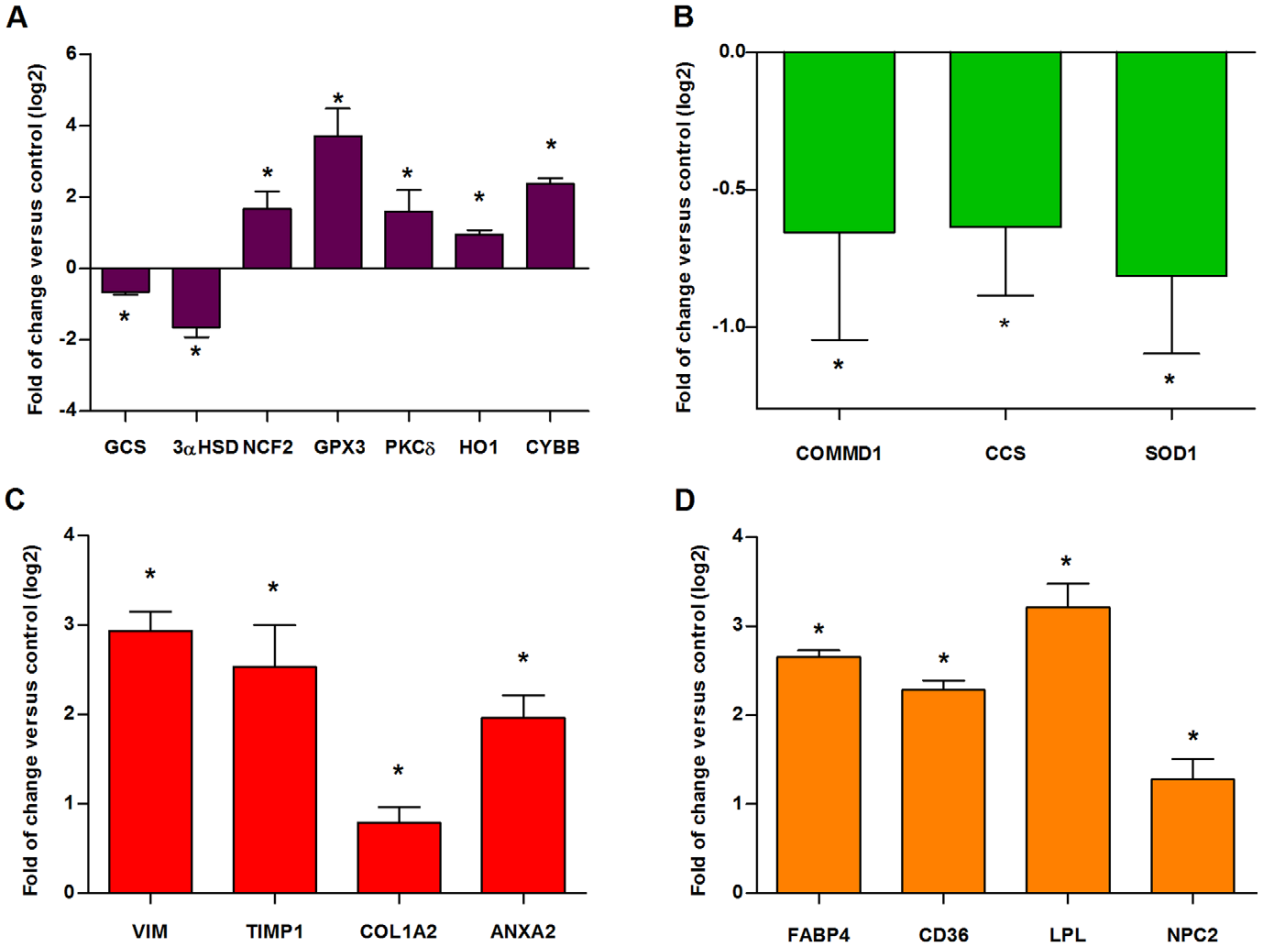
Altered expression profile of genes divided into four functional categories in WT and NPC mice liver. (A) Oxidative Stress; purple bars, (B) Copper Metabolism; green bars, (C) Fibrosis and Inflammation; red bars and (D) Cholesterol Metabolism; orange bars. Hepatic transcript levels of genes were determined by qPCR in WT and NPC mice. For each gene, the transcript level was normalized to *Ppia* in the corresponding sample. qPCR was performed in samples from at least three mice. The mean fold-change is shown as the ratio between WT and NPC mice calculated from two technical replicates. For clarity, data are expressed on a log_2_ scale. Asterisks indicate significant differences between WT and NPC mice (Mann–Whitney U-test, *p<0.05).

We analyzed by qPCR several genes (Table S1) that were expected to be altered in their expression profile due to their function in processes related to NPC in the liver. Figure 3 shows the qPCR results of the genes analyzed. Significant increases in the expression of most of these genes were observed, and we classified the genes into four functional groups: Oxidative Stress (Figure 3A), Copper Metabolism (Figure 3B), Fibrosis and Inflammation (Figure 3C), and Cholesterol Metabolism (Figure 3D).

We observed a significant upregulation (p<0.05, Mann–Whitney U-test) of several genes, including neutrophil cytosolic factor 2 (*Ncf2*), *Gpx3*, protein kinase C delta (*Prkcd*), heme oxygenase 1 (*Hmox1*) and cytochrome b-245, beta polypeptide (*Cybb*) (Figure 3A); vimentin (*Vim*), tissue inhibitor of metalloproteinase 1 (*Timp1*), collagen type I alpha 2 (*Col1a2*) and annexin A2 (*Anxa2*) (Figure 3C); and fatty acid binding protein 4 (*Fabp4*), CD36 antigen (*Cd36*), lipoprotein lipase (*Lpl*) and Niemann Pick type C2 protein (*Npc2*) (Figure 3D). We also observed genes that were significantly downregulated (p<0.05, Mann–Whitney U-test), including glutamate-cysteine ligase (*Gcs*) and 3-alpha hydroxysteroid dehydrogenase (*Dhrs9*) (Figure 3A); and copper metabolism gene MURR1 domain (*Commd1*), copper chaperone for superoxide dismutase (*Ccs*) and superoxide dismutase (*Sod1*) (Figure 3B).

**Figure 4:**
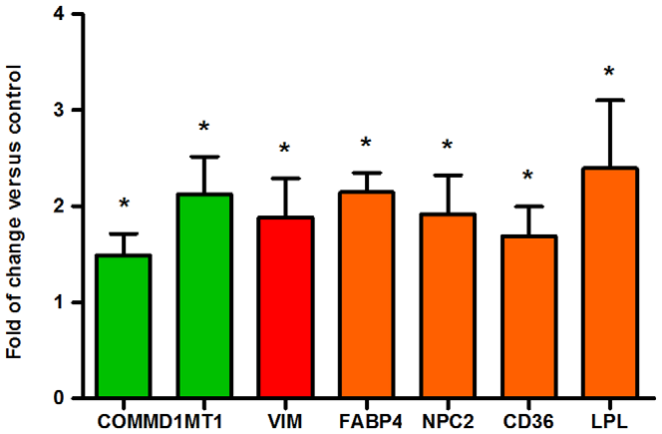
Altered expression profile of several genes in WT and NPC mice cerebellum. Copper Metabolism (green bars), Fibrosis and Inflammation (red bars) and Cholesterol Metabolism (orange bars). Cerebellar transcript levels of genes were determined by qPCR in WT and NPC mice. For each gene, the transcript level was normalized to *Ppia* in the corresponding sample. qPCR was performed in samples from three-five mice. The mean fold-change is shown as the ratio between WT and NPC mice calculated from two technical replicates. Asterisks indicate significant differences between WT and NPC mice (Mann–Whitney U-test, *p<0.05).

We have previously reported [13] an upregulation of several genes involved in oxidative strtess response in cerebellum of NPC mice. Therefore, here we analyzed the expression levels of genes from the other three categories. In this regard, we also found an upregulation of several genes related with cholesterol metabolism (*Cd36, Lpl, Fabp4 and Ncp2; orange bars*) but in only one involved with inflammation and fibrosis (*Vim; red bar*). In the last category, Copper Metabolism, we found instead an up regulation of *Commd1* and also of Metalothioneine 1(*Mt1*; green bars) (Figure 4).

Finally, to obtain a general view of the hepatic gene expression profile in NPC mice, we performed oligonucleotide microarrays to analyze the expression levels of 25,000 genes from NPC mice. We compared three liver samples or four cerebellum samples from 7-week-old NPC mice to a common reference sample generated from pooled mRNA from three WT mice of the same age, for liver samples and four WT mice for cerebellum samples. To screen the microarray data, we initially selected genes that were up- or downregulated at least 2-fold (on the log_2_ scale) and had a p-value of <0.01. We also specifically selected genes that were involved in pathways affecting NPC pathology. The data indicate that regards with liver expression 473 genes were differentially expressed between NPC and WT mice, corresponding to ∼2% of the genes present in the microarray. Among these genes, 282 were up-regulated and 191 were down-regulated in NPC mice compared to WT mice (Table S3). In agreement with the qPCR results, we found an increase in the relative abundance of *Vim*, *Ncsf2*, *Lpl*, *Fabp4* and *Cd36*. Meanwhile, about cerebellum expression, 1325 genes were differentially expressed between NPC and WT mice, corresponding to ∼5.6% of the genes present in the microarray. Among these genes, 328 were up-regulated and 997 were down-regulated in NPC mice compared to WT mice (Table S4). Also, in agreement with the qPCR results, we found an increase in the relative abundance of *Vim*, *Lpl*, *Fabp4* and *Cd36*. Additionally, we performed a GO analysis and found that genes in categories related to cell adhesion, carbohydrate binding, membrane part and oxidoreductase activity were enriched (p<0.05) in hepatic tissue of NPC mice (Table 1 and in more details Table S5) and in cerebellar tissue we found an enrichment of the lymphocyte homeostasis category for up regulated genes and an enrichment of categories related with axonic and synaptic activity for down regulated genes (Table 2 and in more details Table S6).

**Table 1:** Significantly enriched GO categories for differential expressed genes in the liver.

Up-regulated	Down-regulated
Cell adhesion (19)	Molecular transducer activity (38)
Alkane 1-monooxygenase activity (2)	Membrane part (62)
Carbohydrate binding (12)	
Membrane part (8)	

Shown are Gene Ontology (GO) categories with *P*≤0.05 for up and down regulated genes. Numbers in parentheses are numbers of genes in the GO category.

**Table 2:** Significantly enriched GO categories of the differentially expressed genes in the cerebellum.

Up-regulated	Down-regulated
Lymphocyte homeostasis (5)	Protein polymerization (16)
Extracellular region (37)	Regulation of cytoskeleton organization (16)
	Axonogenesis (26)
	Synaptic transmission (30)
	Axon cargo transport (7)
	ATP synthesis coupled proton transport (11)
	ATPase activity coupled to transmembrane movement ions (12)
	Synaptic vesicle (24)

Shown are Gene Ontology (GO) categories with *P≤*0.05 for up and down regulated genes. Numbers in parentheses are numbers of genes in the GO category.

### Acute treatment with N-Acetyl Cysteine of NPC and WT mice

Additionally, we performed a preliminary acute intervention in WT and NPC mice with the antioxidant N-Acetyl Cysteine (NAC) (Text S1). Accordingly to histological analysis (Figure S2), we found that increasing antioxidant levels leads to a partial decrease of the inflammation process, as evidenced by less foamy cells (bold arrows) in the NAC treated NPC mice. Fibrosis, evidenced as collagen deposition (arrow heads), is still present in NPC mice after two weeks NAC treatment. This evidence reinforces the idea that oxidative stress may mediate, in some extension, the hepatic tissue damage observed in this murine model of NPC mice.

In parallel, we also analyzed the possible effect on cerebellar damage (Text S1). We performed immunofluorescence for inflammatory (CD68) and oxidative stress (NITT and HNE) markers in fixed tissue sections of WT, NPC and NPC NAC treated mice (Figure S3). We observed a slight tendency to decrease for CD68 positive signal in NPC NAC treated compared to NPC mice (b–c). We did not find a protection after NAC treatment on oxidative stress, since positive signals for NITT and HNE were not decreased. Taken together these results after acute NAC treatment, we infer that oxidative stress damage is present in both tissues but in cerebellum may begin earlier or faster than in the liver. In that scenario, earlier, longer or stronger (higher doses) NAC treatments could be necesary to get a protective affect at cerebellar level.

## Discussion

NPC is a fatal lysosomal disease where several lipids but mainly free cholesterol is accumulated inside the lysosomes. NPC patients die as a consequence of rapid neurodegeneration. There are several reports indicating that between 45% and 65% of NPC patients also develop hepatic alterations, including cholestasis, jaundice and hepatosplenomegaly [35].

In the infantile form of the disease, hepatic damage resembles neonatal hepatitis but without the presence of Kupffer cells. In the present work, we clearly show the presence of foamy cytoplasm cells, in livers of NPC mice, whereas these cells were not detected in WT livers. In less severe forms of the disease, a progression in hepatic damage is observed along with structural alterations in all of the hepatic cell types. However, the pathogenic mechanisms responsible for the observed hepatic damage are still matter of controversy [18], [36], [37]. In this scenario, is important to highlight our results with an acute NAC intervention in NPC mice (Text S1). The partial decrease observed in the inflammation process after NAC treatment supports the idea that oxidative stress may participate in liver tissue damage. Furthermore, we did not observe any significant change in inflammatory or oxidative stress markers in cerebellum of NPC mice after NAC treatment. This fact suggests that oxidative damage at hepatic level can be more easily reverted or treated than at cerebella. Perhaps, oxidative damage at cerebellum begins earlier in the pathology so, longer and/or earlier NAC treatments should be tested.

Fibrosis has been described in cases of severe hepatic damage. This phenomenon is a response to sustained hepatic damage and is characterized by an increase in extracellular matrix deposition with a notorious increase in production of collagen types I and III. The molecules related to oxidative stress can modulate the cellular events that are responsible for liver fibrosis. Among these molecules, reactive aldehydes formed after lipid peroxidation are especially relevant because they can modulate signal transduction pathways involved in proliferation [38].

In agreement with previously reported data [37], here we show evidence of moderate collagen accumulation in liver of NPC mice. There is no reported evidence of advanced fibrosis in NPC mice. This lack of evidence is probably due to diminished lifespan as a consequence of the neurological alterations, as mice usually die before clear evidence of fibrosis is observed, although advanced hepatic damage is detected [18].

Of note, the gene expression profile presented here is consistent with the histological analysis of NPC livers. The fibrosis shown by collagen deposition and the presence of Kupffer cells can be explained by increased expression of certain molecules, such as ANXA2, VIM, COL1A2 and TIMP1 (for details see Text S1). Current evidence suggests that the process of hepatic fibrosis is driven primarily by an inflammatory reaction in response to parenchymal injury. In agreement with this hypothesis, deletion of specific components of the inflammatory response modifies or attenuates the fibrotic process *in vivo*. In the NPC mouse model, our microarray data support this idea because cell adhesion was one of the most represented categories (n = 19), with several of these genes related to the immune response. Recently, global gene expression analysis of NPC (*Npc1* ^−/−^) mice showed that genes encoding proteins involved in cell adhesion were altered in the cerebellum of 3 week-old animals, suggesting that the absence of NPC1 in the liver induces a compensatory mechanism intended to preserve liver tissue architecture and function [39].

The decrease in GSH and the increase in carbonyls observed in NPC mice suggest diminished antioxidant defenses and the presence of oxidative stress damage. An acute rise in ROS could explain the decrease in GSH, which in turn can enhance the oxidative damage, as evidenced by the increased levels of carbonyls. Notably, ROS can activate several pro-inflammatory and pro-apoptotic pathways [40]. The changes observed in the molecules responsible for antioxidant defense are in agreement with the diminished levels of GSH. Clearly, this result could be explained by increases in HO-1, GPX3 and NCF2 and decreases in GCS and 3αHSD expression levels. We also found upregulation of other oxidative stress–induced genes, such as *Prkcd* [41] and *Cybb* (also named Nox2, a phagocyte NADPH oxidase; for further details see Text S1). CYBB generates ROS in a highly regulated fashion under physiological conditions, but in disease states, its deregulation contributes to oxidative stress and subsequent tissue damage [42].

In summary, the data presented here along with previous work in several other labs support the hypothesis that oxidative stress processes could be mediating the hepatotoxicity in NPC. Furthermore, it has been reported that in hepatocytes from NPC mice, there is an accumulation of cholesterol in mitochondria and a decrease in mitochondrial GSH content [43], and inflammation and apoptosis induced by NPC1 deficiency is significantly reduced in TNFα KO mice [44]. In addition, recently published data show diminished antioxidant capacity in hepatocytes from human NPC patients [21].

With regard to copper metabolism in this NPC model, we actually found an increase in total copper in liver of NPC mice. The copper increase along with the downregulation of SOD1, CCS and COMMD1 could be part of the mechanism responsible for the hepatotoxicity observed in our NPC model and may enhance the damage induced by oxidative stress. The reduction in CCS, the main chaperone of SOD1, which was also downregulated in our model, can be considered a marker for increasing copper content. In fact, in mouse and rat models of reduced dietary copper, CCS expression is augmented [45], and in humans, *Ccs* and *Sod1* mRNA levels are reportedly reduced after dietary supplementation with copper [46]. In agreement with these findings, the reduced expression of COMMD1, which modulates ATP7B localization, would lead to deregulation of copper homeostasis, thereby increasing the damage induced by copper in this disease model. ATP7B is involved in biliary secretion of copper [47], and its malfunction is responsible for Wilson disease.

Our observations about cholesterol and copper metabolism are particularly relevant in NPC liver pathology, as some recent data show that a human hepatoma cell line treated with U18666A, mimicking the NPC phenotype was defective not only in cholesterol trafficking but also in copper secretion. These alterations in copper secretion are associated with intracellular accumulation of copper, suggesting that the function of NPC proteins may be related to ATP7B, a copper-transporting P-type ATPase expressed in hepatocytes [48]. Furthermore, the same group has reported almost complete localization of ATP7B in lysosomes/late endosomes hybrid organelles after U18666A treatment [49].

The data presented here suggest that an imbalance in intracellular cholesterol levels in NPC causes an alteration in intracellular transduction and/or cell adhesion processes. This alteration could lead to an increase in oxidative damage either by enhancing ROS or by decreasing antioxidant species. Damage induced by oxidative stress, especially that mediated by the intracellular accumulation of copper, could be at least one of the mechanisms involved in cellular toxicity observed in NPC pathology. Such alterations in cellular function may be relevant not only to hepatotoxicity but also to neuronal toxicity, mainly in the cerebellum, in NPC. Althought, the exact mechanism that correlates cholesterol accumulation with the oxidative stress response remains unclear, we can speculate that the diminished antioxidant defense along with copper intracellular accumulation and alterations in mitochondrial and peroxisomal function due to cholesterol accumulation [50], [51] may contribute to oxidative stress damage observed in NPC liver pathology as well as in cerebellum. In this regard, recent evidence, including data from our group, show vitamin E accumulation in NPC lysosomes [52], [53], suggesting that a decrease in its bioavailability could contribute to NPC oxidative stress and pathology.

On the other hand, we also analyze the alterations in gene expresion profile of cerebellum, which may account for neurodegeneration processes observed in the pathology. As was expected, genes related with fibrosis were nearly unaffected; meanwhile cholesterol metabolism genes were up-regulated as well as in the liver. Considering global expression analysis we detected enrichment mainly in categories involved in axon and synaptic function. This is quite different respect to what has been previously reported by Liao et al [39] although they analyzed 3 weeks old animals, when neurological symptoms do not appear yet, instead of 7 weeks old when neurological alterations are evident. These two different scenarios in gene expression profile depending on the tissue analyzed, may reflect the differences observed at different stages of the disease progression and/or could be a possible explanation for differential clinical manifestations depending on the age of onset.

Finally, we can conclude that in this NPC mouse model, which recapitulates human NPC, fibrotic liver damage characterized by collagen accumulation and the appearance of foamy cytoplasm cells could be triggered by an increase in oxidative stress. Conditions indicative of an oxidative stress response, such as diminished levels of GSH and augmented levels of carbonyls, can be correlated with the changes observed in gene expression, especially in genes involved in oxidative stress, cholesterol trafficking and copper metabolism. Further studies are required to elucidate how these changes contribute to the pathophysiology of Niemann-Pick Type C disease and the mechanisms involved in oxidative stress in NPC liver.

## Supporting Information

Figure S1 **Selection of the appropriate normalization gene.** (A) Number of qPCR cycles needed for amplification of the gene in the samples studied (7- and 8-week-old WT and NPC mice). Black bar, *Tbp*; gray bar, *Rpl4*; white bar, *Ppia*. (B) Variation coefficient for the three housekeeping genes for each sample studied. Rhomb, *Tbp*; square, *Rpl4*; triangle, *Ppia*.
(TIF)

Figure S2 **Hepatic tissue damage is diminished after acute NAC treatment in NPC mice.** Hematoxylin and Eosin staining (a–d, 20×; e–h, 40×) to assess tissue integrity and Van Gieson staining for collagen (i–l, 40×) in WT (first (saline) and second (NAC) rows) and NPC (third (saline) and fourth (NAC) rows) mice are shown. Inflammatory foci (thin arrows), foamy cytoplasm cells (bold arrows) and fibrosis (arrowheads) are indicated.
(TIF)

Figure S3 **Cerebellar inflammation and oxidative stress damage were not prevented after acute NAC treatment in NPC mice.** CD68 immunofluorescence (a–c) for astrocyte activation assessment, nitrotyrosinilated proteins (NITT; d–f) and 4-Hydroxinonenal adducts (HNE;g–i) for oxidative stress damage visualization in WT, NPC and NPC NAC treated mice. The three markers are increased in NPC compared to WT mice but they are not significantly decreased after NAC treatment in NPC mice.
(TIF)

Table S1 **Genes and gene-specific primers used for the real-time PCR.**
(DOC)

Table S2 **Selection of housekeeping genes (HKGs) for normalization.**
(DOC)

Table S3 **Differentially expressed genes between NPC and WT mice in the liver.**
(XLS)

Table S4 **Differentially expressed genes between NPC and WT mice in the cerebellum.**
(XLS)

Table S5 **Differentially expressed genes in the liver grouped by GO categories**
(XLS)

Table S6 **Differentially expressed genes in the cerebellum grouped by GO categories.**
(XLS)

Text S1 (DOC)
