# Supplementary figures and images for: Alteration of Gene Expression Profile in Niemann-Pick Type C Mice Correlates with Tissue Damage and Oxidative Stress
Source: PLoS One. 2011 Dec 22;6(12):e28777. doi: 10.1371/journal.pone.0028777 (PMC3245218; doi:10.1371/journal.pone.0028777)

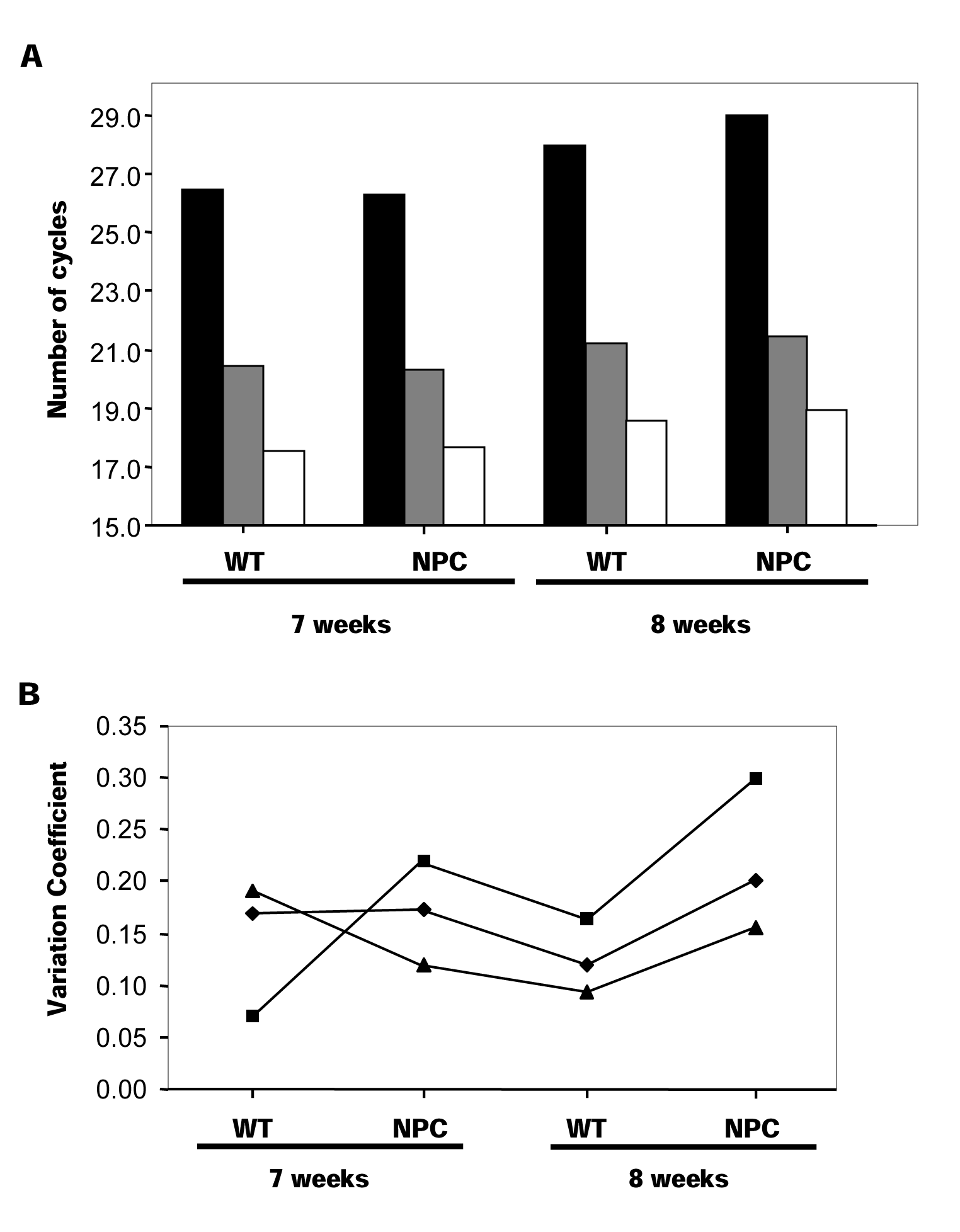

Supplement: Figure S1 — Selection of the appropriate normalization gene. (A) Number of qPCR cycles needed for amplification of the gene in the samples studied (7- and 8-week-old WT and NPC mice). Black bar, Tbp; gray bar, Rpl4; white bar, Ppia. (B) Variation coefficient for the three housekeeping genes for each sample studied. Rhomb, Tbp; square, Rpl4; triangle, Ppia. (TIF) [file pone.0028777.s001.tif]

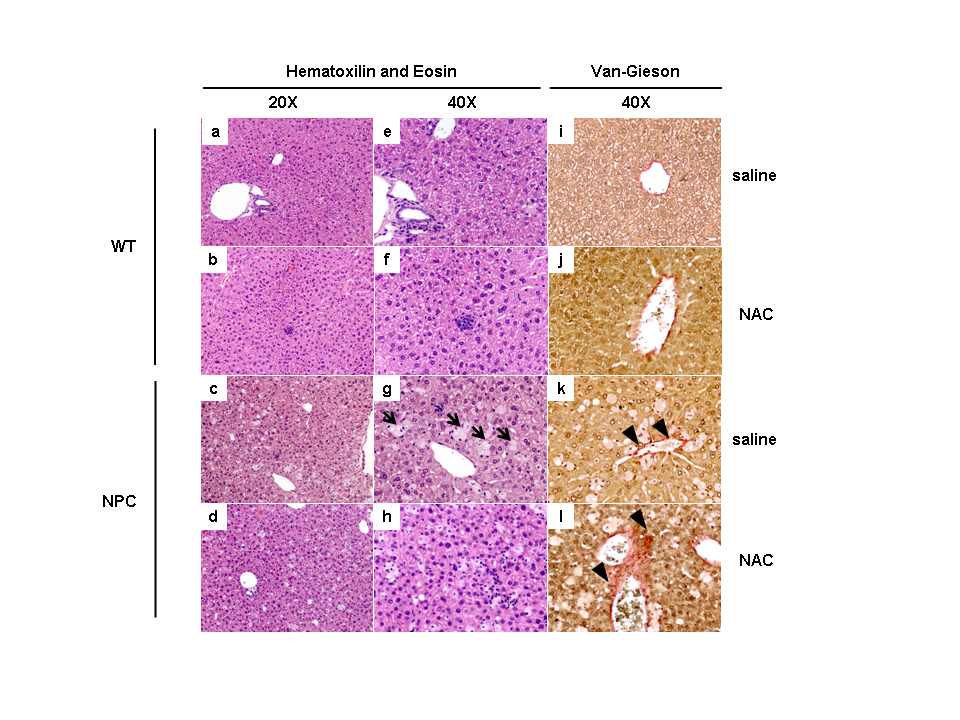

Supplement: Figure S2 — Hepatic tissue damage is diminished after acute NAC treatment in NPC mice. Hematoxylin and Eosin staining (a–d, 20×; e–h, 40×) to assess tissue integrity and Van Gieson staining for collagen (i–l, 40×) in WT (first (saline) and second (NAC) rows) and NPC (third (saline) and fourth (NAC) rows) mice are shown. Inflammatory foci (thin arrows), foamy cytoplasm cells (bold arrows) and fibrosis (arrowheads) are indicated. (TIF) [file pone.0028777.s002.tif]

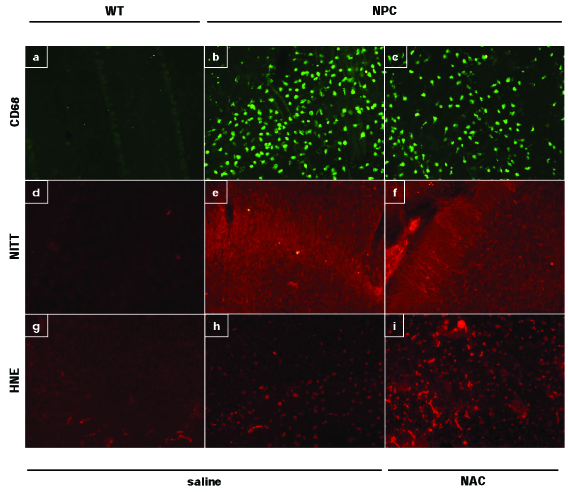

Supplement: Figure S3 — Cerebellar inflammation and oxidative stress damage were not prevented after acute NAC treatment in NPC mice. CD68 immunofluorescence (a–c) for astrocyte activation assessment, nitrotyrosinilated proteins (NITT; d–f) and 4-Hydroxinonenal adducts (HNE;g–i) for oxidative stress damage visualization in WT, NPC and NPC NAC treated mice. The three markers are increased in NPC compared to WT mice but they are not significantly decreased after NAC treatment in NPC mice. (TIF) [file pone.0028777.s003.tif]
