# Supplementary material for: Alteration of Gene Expression Profile in Niemann-Pick Type C Mice Correlates with Tissue Damage and Oxidative Stress
Source: PLoS One. 2011 Dec 22;6(12):e28777. doi: 10.1371/journal.pone.0028777 (PMC3245218; doi:10.1371/journal.pone.0028777)
Supplement: Table S1 — Genes and gene-specific primers used for the real-time PCR. (DOC) [file pone.0028777.s004.doc]

**Table S1. Genes and gene-specific primers used for the real-time PCR.**

| **Gene NCBI (ID) Forward primer (5'→ 3') Reverse primer (5'→ 3') Product** |
| --- |

| PPIA | DQ985731.1 | GTGGTCTTTGGGAAGGTG | GGTGATCTTCTTGCTGGTC | peptidylprolyl isomerase A |
| --- | --- | --- | --- | --- |
| RPL4 | NM_024212.4 | CCAGAAATCACAAACTTCGTG | CTTTCTTGCCTACCGCTG | ribosomal protein L4 |
| TBP | NM_013684.3 | GCTGTAAACTTGACCTAAAGAC | TCTTCACTCTTGGCTCCT | TATA box binding protein |
| VIM | NM_011701 | AAGCAGGAGTCAAACGAG | TTCCATCTCACGCATCTG | vimentin |
| ANXA2 | NM_007585 | GAAGGACATCATCTCTGACAC | GTAGTCAATAACTGAGCCATCC | annexin A2 |
| TIMP-1 | NM_011593 | ATCCTCTTGTTGCTATCACTG | GGTCTCGTTGATTTCTGGG | tissue inhibitor of metalloproteinase 1 |
| COL1A2 | NM_007743 | TTAAGACTCAGCCACCCA | TCACCAGTAGAGAAATCACAG | collagen, type I, alpha 2 |
| FABP4 | NM_024406 | GAAATCACCGCAGACGAC | TTCATAACACATTCCACCACC | fatty acid binding protein 4, adipocyte |
| NCF2 | NM_010877 | CTTCGGATTCACCCTCAG | CTTCCTTCAGTTCCTTGGGCTC | neutrophil cytosolic factor 2 |
| CYBB | NM_007807 | CCCATTCACACTGACCTC | CTGCTATCTTAGGTAGTTTCCA | cytochrome b-245, beta polypeptide |
| GPX3 | NM_008161 | CATTTGGCTTGGTCATTCTG | CGAACATACTTGAGACTGGG | glutathione peroxidase 3 |
| LPL | NM_008509 | TGAGTTGTAGAAAGAATCGCTG | TGGTAATGGAACACTTTGTAGG | lipoprotein lipase |
| CD36 | NM_007643 | GGCTAAATGAGACTGGGAC | GCAACAAACATCACCACTC | CD36 antigen |
| DHRS9 | NM_134072 | TGATTGCCATTCGTTACCA | ACTCTTTGATCCGCTTCTC | 3-alpha hydroxysteroid dehydrogenase |
| GCS | NM_010295 | GGGGTGACGAGGTGGAGTA | GTTGGGGTTTGTCCTCTCCC | glutamate-cysteine ligase, catalytic subunit |
| HO1 | NM_010442 | CACAGCACTATGTAAAGCGTCT | TGTGCAATCTTCTTCAGGACC | heme oxygenase 1 |
| PKCd | NM_011103 | CGACATGCCTCACCGATTCAAGG | GTTGGCCACCTTCTCCCGGC | protein kinase C, delta |
| COMMD1 | NM_144514 | CGCAGAACGCCTTTCACGG | TTTGCTTGACTTTAACTTCATC | copper metabolism gene MURR1 domain |
| CCS | NM_016892 | TCCCTTATCCAAGATCACAG | CGAGGACCAAATAACCTGA | copper chaperone for superoxide dismutase |
| SOD1 | NM_011434 | GAAGCATGGCGATGAAAGC | ACTGATGGACGTGGAACCC | superoxide dismutase 1, soluble |
| NPC2 | NM_023409 | TATCTTGTGACTGCTCGG | CTGGTAAAGGTGATGTTGA | Niemann Pick type C2 |
