# Supplementary material for: Alteration of Gene Expression Profile in Niemann-Pick Type C Mice Correlates with Tissue Damage and Oxidative Stress
Source: PLoS One. 2011 Dec 22;6(12):e28777. doi: 10.1371/journal.pone.0028777 (PMC3245218; doi:10.1371/journal.pone.0028777)
Supplement: Table S2 — Selection of housekeeping genes (HKGs) for normalization. (DOC) [file pone.0028777.s005.doc]

**Table S2. Selection of housekeeping genes (HKGs) for normalization.**

| **Gene Symbol** | **Stability Value** |
| --- | --- |
| TBP | 0.478 |
| RPL4 | 0.219 |
| PPI | 0.136 |
