# Supplementary material for: Alteration of Gene Expression Profile in Niemann-Pick Type C Mice Correlates with Tissue Damage and Oxidative Stress
Source: PLoS One. 2011 Dec 22;6(12):e28777. doi: 10.1371/journal.pone.0028777 (PMC3245218; doi:10.1371/journal.pone.0028777)
Supplement: Text S1 — (DOC) [file pone.0028777.s010.doc]

**SUPPLEMENTARY DATA**

SUPPLEMENTARY MATERIALS AND METHODS

***Animals’ treatment:***

For NAC treatments, between P42 and P56, WT and NPC mice were daily injected by i.p., with either saline solution (NaCl 0,9% in water) or N-acetylcisteine 20mg/mL diluted in saline solution from a 100 mg/mL comercial solution (Instituto Sanitas, Santiago, Chile). The dose was 100 mg/kg. Control animals received an equivalent volume of saline solution. At the end of the treatment animals were sacrificed and hepatic and cerebellar tissue were dissected for histological analysis and serum was obtained for alanine aminotransferase (ALAT) measurement by ALAT kit (Kovalent, Rio de Janeiro, Brazil) following manufacturer’s instructions

***Immunofluorescence:***

Sections to measure immunofluorescence were incubated over night with rat anti-mouse CD68 (MCA 1957GA, 1:1000, AbD Serotec, Oxford, UK), rabbit anti-mouse Nitrotyrosine (NITT12-A,1:1000, Alpha Diagnostic, San Antonio, TX, USA), goat anti-mouse 4 Hydroxynonenal (AB5605,1:100; Chemicon, Harrow, UK). Secondary antibodies used were goat anti-rat conjugated with FITC (81-9511, 1:100; Zymed, San Francisco, CA., USA), goat anti-rabbit Alexa 555 (A21422, 1:2000; Invitrogen, Carlsbad, CA, USA) and rabbit anti-goat rhodamine conjugated (03-13-06, 1:2000; KPL, Gaithersburg, MD, USA) respectively.

SUPPLEMENTARY RESULTS

As a preliminary acute intervention in WT and NPC mice with the antioxidant NAC, we injected intraperitonealy with daily frequence WT and NPC mice for a two weeks period. After treatment, animals were sacrificed and hepatic tissue was stained with Hematoxilin and Eosin to evaluate tissue integrity and with Van Gieson to assess collagen deposition, as fibrosis indicator (Figure S2). We found less foamy cytoplasm cells (bold arrows) in NAC treated NPC mice (d,h) as well as a tendency to decrease of inflammation foci (thin arrow), when compared with saline NPC mice (c,g). In addition, a tendency to decrease of the liver damage-related marker ALAT was detected in serum of NAC treated NPC mice (205.5 ± 17.7 U/L vs 241.7 ± 144.9 U/L in NAC treated and untreated NPC mice).

We did not observe a significant change respect to fibrosis (collagen deposition, arrow heads) in NPC mice after two weeks NAC treatment. In WT mice we did not evidencied any significant difference betwen saline (a,e and i) and NAC treated (b, f and j) animals, indicating that i.p injection itself did not affect tissue integrity significantly.

Regards to the possible effect at the cerebellum, we did not find any significant improvement after NAC treatment in NPC mice. Positive immunofluorescent mark for CD68 (a-c) shows just a slight tendency to decrease in this inflammation marker in NPC NAC treated mice (b compared to c) and NITT and HNE both oxidative stress markers are not decreased in NPC NAC treated mice. These results suggest that acute NAC treatment is not enough to reverse the inflammatory and oxidative processes in the cerebellum. Longer or earlier treatment times should be tested in the future to address this issue.

SUPPLEMENTARY DISCUSSION

Regard to gene expression changes in hepatic tissue it is important to note that we found an increase in several genes related to fibrosis and inflammation processes, which is correlated with the evidence provided by the histological analysis. These up-regulated genes were *Anxa2*, *Vim*, *Col1a2* and *Timp1*. ANXA2 is a calcium-dependent phospholipid-binding protein that may be involved in proliferative processes as a recovery mechanism after tissue damage **[1]**. VIM is a type III intermediate filament protein **[2]**, and COL1A2 is an extracellular matrix component that has been used to study the transcriptional control of extracellular matrix deposition under normal and fibrotic conditions and serves as a biological marker for fibrotic processes **[3]**. Finally, we found upregulation of *Timp1*, encoding a molecule that can further increase collagen I levels by inhibiting its degradation by collagenase; TIMP1 is also known to be augmented in fibrotic processes **[4]** by a mechanism likely involving activation of hepatic stellate cells by TGFβ, which is released by Kupffer cells **[5]**

Continuing the gene expression analysis of the liver, we also found a significant increase in genes involved in cholesterol metabolism, including *Lpl*, *Fabp4*, *Cd36* and *Npc2*, in accord with previous reports. Although FABP4 is considered adipocyte-specific, it is increased in fatty livers **[6]** and is also expressed in macrophages. In fact, FABP4 expression increases during macrophage activation **[7]**, and this correlates with the presence of foam cells observed by hematoxylin and eosin staining. As was previously described, the scavenger receptor CD36, which is involved in lipid uptake by internalization of oxidized LDL and foam cell formation **[8]**, was increased in our model, as was NPC2, probably as a mechanism to compensate for the lack of NPC1 expression **[9]**. We found decreased expression of 3αHSD (3-alpha hydroxysteroid dehydrogenase), which is involved in bile acid biosynthesis as well as steroid hormone and xenobiotic metabolism **[10]**. There is some evidence suggesting the involvement of 3αHSD in hormonal regulation **[11]**, but it has also been reported that hepatic activity does not depend on gender **[12]**. In any case, we performed our analysis only in males.

Going further, after GO analysis of liver microarrays, an interesting example of one of the upregulated genes was *Gpnmb* (osteoactivin), which Haralanova-Ilieva et al. **[13]** demonstrated is expressed at high levels in normal and inflammatory liver macrophages, suggesting a role for this protein in acute liver injury. More recently, Abe et al. **[14]** showed that transgenic rats expressing osteoactivin exclusively in hepatocytes exhibit attenuated hepatic fibrosis in response to a diet that induces liver steatosis. The expression of the cell adhesion molecule VCAM1 is confined to hepatic stellate cells (HSCs), which are key to the process of fibrogenesis **[15]**. TGF-beta2 signaling, particularly beta2-Spectrin, has a critical function in hepatocyte proliferation and transitional phenotype, and the loss of TGF-beta2 is associated with activation of hepatic progenitor cells secondary to delayed mitogenesis and activated Wnt signaling **[16]**. We also found other genes involved in cell adhesion processes, that were upregulated in NPC versus WT mice, such as the *Gal-1*, embigin, *Bpag1* (bullous pemphigoid antigen 1) and alpha-catenin genes. The increased expression of GAL-1, which is a member of a family of lectins, has been observed in several malignant tumors, including hepatocellular carcinoma **[17]**. Embigin, a glycoprotein with two immunoglobulin-like domains, is preferentially expressed in the early stages of mouse embryogenesis and enhances integrin-mediated cell-substratum adhesion **[18]**. The expression of embigin is required to maintain the transporter activity of monocarboxylic acid transporter 2, which is important for the uptake of lactic acid for gluconeogenesis in the liver **[19]**. BPAG1 is a component of the hemidesmosomes that can bind to intermediate filaments **[20]** and alpha-catenin is a member of adherens junctions that connect cadherin complexes to the actin cytoskeleton **[21]**. These findings support the concept that hepatic tissue damage is relevant during NPC disease progression.

SUPPLEMENTARY REFERENCES

1. Cheng CW, Rifai A, Ka SM, Shui HA, Lin YF, et al. (2005) Calcium-binding proteins annexin A2 and S100A6 are sensors of tubular injury and recovery in acute renal failure. Kidney Int 68: 2694-2703.

2. Lopez-Egido J, Cunningham J, Berg M, Oberg K, Bongcam-Rudloff E, et al. (2002) Menin's interaction with glial fibrillary acidic protein and vimentin suggests a role for the intermediate filament network in regulating menin activity. Exp Cell Res 278: 175-183.

3. Ramirez F, Tanaka S, Bou-Gharios G (2006) Transcriptional regulation of the human alpha2(I) collagen gene (COL1A2), an informative model system to study fibrotic diseases. Matrix Biol 25: 365-372.

4. Arthur MJ (2000) Fibrogenesis II. Metalloproteinases and their inhibitors in liver fibrosis. Am J Physiol Gastrointest Liver Physiol 279: G245-249.

5. Cao Q, Mak KM, Lieber CS (2002) Dilinoleoylphosphatidylcholine prevents transforming growth factor-beta1-mediated collagen accumulation in cultured rat hepatic stellate cells. J Lab Clin Med 139: 202-210.

6. Westerbacka J, Kolak M, Kiviluoto T, Arkkila P, Siren J, et al. (2007) Genes involved in fatty acid partitioning and binding, lipolysis, monocyte/macrophage recruitment, and inflammation are overexpressed in the human fatty liver of insulin-resistant subjects. Diabetes 56: 2759-2765.

7. Kazemi MR, McDonald CM, Shigenaga JK, Grunfeld C, Feingold KR (2005) Adipocyte fatty acid-binding protein expression and lipid accumulation are increased during activation of murine macrophages by toll-like receptor agonists. Arterioscler Thromb Vasc Biol 25: 1220-1224.

8. Moore KJ, Kunjathoor VV, Koehn SL, Manning JJ, Tseng AA, et al. (2005) Loss of receptor-mediated lipid uptake via scavenger receptor A or CD36 pathways does not ameliorate atherosclerosis in hyperlipidemic mice. J Clin Invest 115: 2192-2201.

9. Klein A, Amigo L, Retamal MJ, Morales MG, Miquel JF, et al. (2006) NPC2 is expressed in human and murine liver and secreted into bile: potential implications for body cholesterol homeostasis. Hepatology 43: 126-133.

10. Penning TM, Burczynski ME, Jez JM, Hung CF, Lin HK, et al. (2000) Human 3alpha-hydroxysteroid dehydrogenase isoforms (AKR1C1-AKR1C4) of the aldo-keto reductase superfamily: functional plasticity and tissue distribution reveals roles in the inactivation and formation of male and female sex hormones. Biochem J 351: 67-77.

11. Stravitz RT, Vlahcevic ZR, Pandak WM, Stolz A, Hylemon PB (1994) Regulation of rat hepatic 3 alpha-hydroxysteroid dehydrogenase in vivo and in primary cultures of rat hepatocytes. J Lipid Res 35: 239-247.

12. Pirog EC, Collins DC (1999) Metabolism of dihydrotestosterone in human liver: importance of 3alpha- and 3beta-hydroxysteroid dehydrogenase. J Clin Endocrinol Metab 84: 3217-3221.

13. Haralanova-Ilieva B, Ramadori G, Armbrust T (2005) Expression of osteoactivin in rat and human liver and isolated rat liver cells. J Hepatol 42: 565-572.

14. Abe H, Uto H, Takami Y, Takahama Y, Hasuike S, et al. (2007) Transgenic expression of osteoactivin in the liver attenuates hepatic fibrosis in rats. Biochem Biophys Res Commun 356: 610-615.

15. Erkan M, Weis N, Pan Z, Schwager C, Samkharadze T, et al. Organ-, inflammation- and cancer specific transcriptional fingerprints of pancreatic and hepatic stellate cells. Mol Cancer 9: 88.

16. Thenappan A, Li Y, Kitisin K, Rashid A, Shetty K, et al. Role of transforming growth factor beta signaling and expansion of progenitor cells in regenerating liver. Hepatology 51: 1373-1382.

17. Spano D, Russo R, Di Maso V, Rosso N, Terracciano LM, et al. Galectin-1 and its involvement in hepatocellular carcinoma aggressiveness. Mol Med 16: 102-115.

18. Tachikui H, Kurosawa N, Kadomatsu K, Muramatsu T (1999) Genomic organization and promoter activity of embigin, a member of the immunoglobulin superfamily. Gene 240: 325-332.

19. Ovens MJ, Manoharan C, Wilson MC, Murray CM, Halestrap AP The inhibition of Monocarboxylate Transporter 2 (MCT2) by AR-C155858 is modulated by the associated ancillary protein. Biochem J.

20. Sonnenberg A, Liem RK (2007) Plakins in development and disease. Exp Cell Res 313: 2189-2203.

21. Weis WI, Nelson WJ (2006) Re-solving the cadherin-catenin-actin conundrum. J Biol Chem 281: 35593-35597.

SUPPLEMETARY FIGURES AND TABLES LEGENDS

**Figure S1. Selection of the appropriate normalization gene**. (A) Number of qPCR cycles needed for amplification of the gene in the samples studied (7- and 8-week-old WT and NPC mice). Black bar, *Tbp*; gray bar, *Rpl4*; white bar, *Ppia*. (B) Variation coefficient for the three housekeeping genes for each sample studied. Rhomb, *Tbp*; square, *Rpl4*; triangle, *Ppia*.

**Figure S2. Hepatic tissue damage is diminished after acute NAC treatment in NPC mice.** Hematoxylin and Eosin staining (a-d, 20X; e-h, 40X) to assess tissue integrity and Van Gieson staining for collagen (i-l, 40X) in WT (first (saline) and second (NAC) rows) and NPC (third (saline) and fourth (NAC) rows) mice are shown. Inflammatory foci (thin arrows), foamy cytoplasm cells (bold arrows) and fibrosis (arrowheads) are indicated.

**Figure S3. Cerebellar inflammation and oxidative stress damage were not prevented after acute NAC treatment in NPC mice.** CD68 immunofluorescence (a-c) for astrocyte activation assessment, nitrotyrosinilated proteins (NITT; d-f) and 4-Hydroxinonenal adducts (HNE;g-i) for oxidative stress damage visualization in WT, NPC and NPC NAC treated mice. The three markers are increased in NPC compared to WT mice but they are not significantly decreased after NAC treatment in NPC mice.

**Table S1. Genes and gene-specific primers used for the real-time PCR.**

**Table S2. Selection of housekeeping genes (HKGs) for normalization.**

**Table S3.** **Differentially expressed genes between NPC and WT mice in the liver.**

**Table S4.** **Differentially expressed genes between NPC and WT mice in the cerebellum.**

**Table S5.** **Differentially expressed genes in the liver grouped by GO categories**

**Table S6.** **Differentially expressed genes in the cerebellum grouped by GO categories.**
